# Perceived Paternal and Maternal Parenting Attributes among Chinese Adolescents: A Meta-Analysis

**DOI:** 10.3390/ijerph17238741

**Published:** 2020-11-24

**Authors:** Diya Dou, Daniel T. L. Shek, Ka Ho Robin Kwok

**Affiliations:** Department of Applied Social Sciences, The Hong Kong Polytechnic University, Hong Kong, China; diya.dou@polyu.edu.hk (D.D.); robin.kwok@polyu.edu.hk (K.H.R.K.)

**Keywords:** parenting, parental difference, maternal parenting, paternal parenting, adolescents, Chinese, meta-analysis

## Abstract

This meta-analysis study examined perceived parental differences between Chinese mothers and fathers from the perspective of adolescents. A systematic search for relevant articles published up to 2019 was performed in electronic databases. The random-effect model was used to calculate the weighted and pooled effect size at the 95% confidence interval. This study was based on 43 studies in English peer-reviewed journals involving 55,759 Chinese adolescents aged between 11 and 18 years. We conducted subgroup analyses to explore whether differences in study designs (i.e., cross-sectional and longitudinal) and adolescent gender could explain perceived parental differences. The results showed that perceived maternal parenting attributes were more positive than perceived paternal parenting attributes in cross-sectional and longitudinal studies. Besides, perceived paternal parenting attributes showed slightly greater variability than did maternal parenting attributes. Subgroup analysis based on adolescent gender revealed that only adolescent girls perceived maternal parenting attributes to be more positive than paternal parenting attributes.

## 1. Introduction

Parents have tremendous influences on various aspects of adolescent development. As a basic family process, positive parenting can promote adolescent school achievement [1], cognitive development [2], and social behaviors [3].

Family research has identified different roles of mothers and fathers in family functioning, which shed light on parenting practices for creating a positive family environment [4]. In Chinese societies, parental roles have been inherently constrained by traditional Chinese cultures and also profoundly shaped by contemporary social and economic development. On the one hand, Chinese fathers have been traditionally expected to lead the family and educate their children properly. Meanwhile, mothers should take care of the family in a subordinate role. On the other hand, the increase in women’s employment and education nowadays may alter the distribution of household and parenting responsibilities [5]. Moreover, some new characteristics of parental behaviors have been increasingly observed in Chinese parents, especially in mothers [6]. As described in terms such as “helicopter parenting” and “tiger mothers,” Chinese mothers may use over-parenting and harshness to push their children to achieve future success [7]. Besides, existing research has revealed equivocal findings on parental differences between mothers and fathers in Chinese societies [5,8,9,10]. To fill the research gap, this meta-analysis study aims to systematically review the literature on parental differences between Chinese mothers and fathers. 

### 1.1. Parental Differences in Parenting

Mothers and fathers play different roles in the family process. According to Bem’s sex-role theory [11], fathers’ parenting behaviors are often goal-oriented, reflecting their masculine instrumental personality traits. In contrast, mothers provide more emotional support as they possess expressiveness traits. Besides, fathers and mothers provide adolescents with different experiences in gender socialization and gender typing [4]. Empirical research has also revealed some commonly shared parental differences across cultures. Compared to fathers, mothers have stronger bonds with children, spend more time in parenting, and are more sensitive and supportive to their children [12]. In contrast, fathers often engage more in energetic play and provide more autonomy relating to challenges [13]. Mothers consistently provide support and warmth to children throughout childhood to adolescence, while fathers’ role in parenting becomes more salient during adolescence [14].

### 1.2. Parental Differences in Chinese Contexts

Despite the commonly observed parental differences, scholars have argued that it is important to understand parental differences in specific cultural contexts [10,15]. This is because social and cultural aspects can significantly influence how parents practice their parenting behaviors, how adolescents perceive these behaviors, and how specific parenting behaviors influence adolescents. In Chinese societies, collectivism plays a profound role in regulating interpersonal relationships. The emphasis on the common interests and interdependency within a group will shape family structure and relationships. Traditional Chinese cultures set clear roles for family members, with men leading the family and educating children properly. As revealed in a Chinese saying, “*zi bu jiao*, *fu zhi guo*”, fathers should take the responsibility to educate children with good characters and manners. By contrast, women should take care of the whole family in a subordinate role. A Chinese saying “*yan fu ci mu*”, which means “strict fathers and kind mothers,” precisely depicts the role expectations of Chinese parents in traditional Chinese cultures.

Empirical research on parenting in Chinese contexts has provided evidence in understanding parental differences. Maccoby and Martin’s two-dimensional framework [16] has been used to organize findings involving different dimensions of parenting. The first dimension is parental responsiveness, referring to “the extent to which parents intentionally foster individuality, self-regulation, and self-assertion by being attuned, supportive, and acquiescent to children’s special needs and demands” [17]. Parental responsiveness covers parental warmth and quality of communication. Previous research has revealed that Chinese mothers are more responsible in comparison to Chinese fathers. For example, Wang’s study on adolescents in Beijing, China, revealed that mothers were perceived to provide higher emotional warmth than fathers [18]. Consistent findings were found in adolescents in Jinan and Shanghai, China [18,19]. Besides, Chinese adolescents perceived higher levels of maternal responsiveness and better mother-child communication than those of fathers [20,21,22,23]. Shek, Lee, and Lam’s study [24] revealed that mother–child communication was higher than father–child communication, based on Hong Kong adolescents. Adolescents recruited from schools in different cities in China consistently perceived significantly better mother–adolescent relationships than father–adolescent relationships [25,26]. A 6-year longitudinal study conducted with Hong Kong adolescents revealed that mother–adolescent relationship qualities were significantly higher than paternal–adolescent relationship qualities across all six waves [27,28,29]. The research has consistently shown that Chinese mothers are perceived to be more responsive, supportive, and caring, and have better relationships with adolescents.

The second dimension is parental demandingness, such as behavioral and psychological control, representing the control factors to adolescents’ development [30]. It refers to “the claims parents make on children to become integrated into the family whole by their maturity demands, supervision, disciplinary efforts and willingness to confront the child who disobeys” [17]. Previous studies have revealed that mothers are more demanding and stricter than fathers [20,21,31]. Research indicated that perceived maternal control was significantly higher than perceived paternal control among adolescent boys and girls in Shanghai, China [32]. Moreover, studies revealed that perceived maternal psychological and behavioral control were higher than those of fathers [27,28,29,33]. Perceived rejection was also found to be higher in Chinese mothers in Shandong province of Eastern China [34]. Similarly, mothers were perceived to use harsher discipline than were fathers in Hong Kong [20] and Beijing [18]. Generally speaking, Chinese mothers have been perceived to be more demanding, controlling, and stricter in comparison to fathers.

Some observations can be drawn based on the above literature review. First, the roles of Chinese parents nowadays are different from the role expectations in traditional cultures. As mentioned earlier, fathers are expected to strictly regulate children’s behaviors according to traditional Chinese cultures. However, contemporary literature has suggested that Chinese mothers are taking dominant roles in educating children. As Shek [35] argued, the traditional Chinese cultural belief of “strict father, kind mother” has shifted to a “strict mother, kind father” picture. Second, the equivocal findings fail to answer the following question: do Chinese adolescents perceive maternal parenting attributes and paternal parenting attributes differently? According to the literature, Chinese mothers possess high levels of perceived positive parenting attributes, such as warmth and responsiveness, and high levels of negative attributes, such as harshness and control. One possible reason concerns the different dimensions and scales used in studies. As argued by Shek [9], different definitions of “strictness,” such as “harshness” or “demandingness,” can lead to conflicting perceptions of adolescents. These observations call for a meta-analysis study that quantitatively synthesizes the evidence for the parental differences between Chinese mothers and fathers.

In the present study, we examined perceived parental differences in Chinese adolescents based on weighted and pooled effect size estimates. We further conducted a subgroup analysis based on study design (i.e., cross-sectional and longitudinal analysis). As suggested by Adachi and Willoughby [36], concurrent correlations revealed in cross-sectional studies might be attributed to reciprocal associations of research variables. Besides, the changes in variables over time may reduce the power of variables. Some empirical longitudinal research also revealed that the levels of parental control and support decreased during adolescence [37]. Therefore, cross-sectional studies and longitudinal studies may reveal distinct patterns of parental differences between mothers and fathers.

In addition, we performed a subgroup analysis by adolescent gender. Parents tend to adopt different parenting behaviors when raising boys and girls [38], particularly in Chinese culture. For example, parents often provide more autonomy for boys, but exercise greater control over girls [39]. According to social learning theory, a child’s development is greatly affected by the same-sex parent [40]. Moreover, in comparison to adolescent boys, girls often perceive better relationships with parents, and thus may have more positive perceptions of parenting attributes [10,37]. Branje [41] also argued that mother–daughter relationships are the closest. Thus, adolescent girls and boys may perceive maternal and paternal parenting attributes differently.

### 1.3. Research Questions and Hypotheses

Based on theoretical considerations and empirical findings, we attempted to answer three research questions. 

Research Question 1: Is maternal parenting perceived to be more positive than paternal parenting? Based on previous research, Chinese adolescents perceived their mothers to be warmer, more responsible, and supportive [10]. Although some negative parenting behaviors of mothers were observed, it is argued that the effects of these negative behaviors may be less detrimental on Chinese adolescents as cultural norms emphasize children’s obligations to the family [42].

**Hypothesis 1** **(H1).** *Perceived maternal parenting attributes would be generally more positive than perceived paternal parenting attributes.*

Research Question 2: Are perceived parental differences consistent in cross-sectional and longitudinal studies? The literature has suggested that parental differences may become less evident over time [36,37].

**Hypothesis 2** **(H2).** *Parental differences would be stronger in cross-sectional studies than in longitudinal studies.*

Research Question 3: Are perceived parenting attributes different in adolescent boys and girls?

**Hypothesis 3** **(H3).** *Adolescent females would perceive maternal parenting more positively than adolescent males would* [40,41].

## 2. Methods 

### 2.1. Identification of Studies 

PsycINFO, Ovid Medline, and Embase electronic databases were searched for articles published in English up to December 2019. As we attempted to find as many studies as possible, a review of the structured and controlled vocabulary thesaurus in each electronic database was performed. The indexing of all journal articles differs between the databases, such as Medical Subject Headings (MeSH) terms in Ovid Medline, Subject Headings in PsycINFO, and Emtree in Embase database. After the review of controlled vocabularies in relating to parenting behavior, the following key terms were used for all databases: parental behavior* OR parenting OR family relation* OR family and parenting measure* OR paternal behavior* OR maternal behavior* OR parental attitude* OR parent child relation* OR father child relation* OR mother child relation* OR parental involvement OR parent child communication OR parental role and China OR Hong Kong OR Chinese.

Several inclusion criteria were used in this meta-analysis. First, studies for inclusion separately measured adolescents’ perceptions of both paternal and maternal parenting attributes. Second, the reviewed studies reported adequate information for effect size computations, such as means and standard deviations, for statistically comparing perceived paternal and maternal parenting attributes. Third, the age range of the participants was from 11 to 18 years, with mean age within the age range was acceptable. Fourth, at least 34 participants were included in the study. For paired *t*-tests that are often used to examine the perceived parental differences, a minimum sample size of 34 is required to achieve a power of 80% and a level of significance of 5% (two-tailed) for detecting an effect size of 0.5 between pairs [43]. Fifth, study participants included Chinese adolescents. Finally, the studies were published in English peer-reviewed journal. We excluded studies that (a) collected parental data from fathers and mothers (but not from adolescents); (b) examined combined parenting attributes without distinguishing mothers and fathers; (c) conducted qualitative studies; (d) did not use measures examining parenting attributes; and (e) did not provide adequate information for coding (e.g., the ethnicity of the sample) and statistical computation.

**Figure 1:**
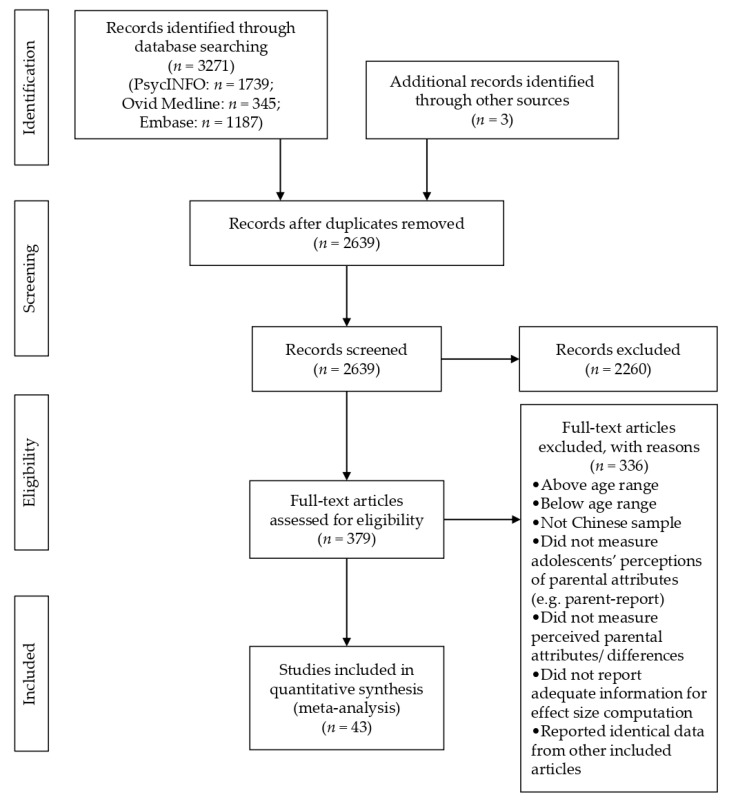
PRISMA flow diagram outlining the selection process.

The review process included several steps. First, abstracts and citations were imported into EndNote version X9 (Clarivate Analytics, London, UK) for screening. All duplicates were removed. Second, based on title and abstract screening, articles were reviewed to be included in the next stage for full-text examination. The reference list of each included study was manually searched and evaluated for inclusion to locate relevant studies that did not found in the electronic databases. There was no disagreement between the researchers on the eligibility of the included studies. The entire screening process was adopted from the PRISMA statement and diagram (Figure 1). Third, full-text articles of all included studies were obtained to evaluate and record the main findings and methodological details. For each included study, the following information was recorded: (a) geographic location; (b) the number of adolescent participants (e.g., by total, subgroups, or time points); (c) mean age of participants or school grade; (d) percentage of female participants; (e) study design; (f) source of reporting information on whether perceived parenting attributes for fathers and mothers reported separately in adolescent males and adolescent females; (g) the measure of perceived parental differences (e.g., questionnaire or inventory) used; (h) the dimensions of parenting attributes examined in the measure; and (i) all statistics concerning adolescents’ perceptions of maternal and paternal parenting attributes, including means and standard deviations. The included studies are shown in Table 1.

### 2.2. Data Analysis

Review Manager version 5.3 [76], a computer software developed by Cochrane (Copenhagen, Denmark) to conduct reviews and meta-analysis studies, was used to calculate the effect size estimates. The approaches for dealing with multiplicity in the outcome domain (multiple dimensions of parenting attributes measured within a study) and time frame (multiple time points measured for a single dimension within a study) are discussed below.

For studies reported multiple dimensions of parenting attributes, each measured dimension was treated as an independent effect size estimate in the analysis of overall effect. For example, support and monitoring that were assessed by a measure in a single study were regarded independently as two separate estimates of effects. Following the Cochrane handbook for systematic reviews [77], for studies reported perceived parenting attributes at multiple time points, only effect size from Wave 1 data was included in the analysis of an overall effect. Studies that reported data for several subgroups, such as year of school (e.g., junior and senior), were combined to calculate the effect size estimates. In all analyses, we only use each effect size once to ensure the independence of each reported observation.

Moreover, the multiple dimensions of parenting attributes included in the studies can be categorized as positive measures (e.g., support and warmth) or negative measures (e.g., rejection and love withdrawal). To manage the effect direction, each measured dimension was evaluated and categorized into positive or negative parenting attributes. Disagreements were resolved by discussion among the researchers until a consensus was reached. Negative parenting attributes were reverse-coded into the same direction as positive parenting attributes. Therefore, higher scores reflected more positive parenting characteristics.

Furthermore, we performed additional subgroup meta-analyses by study design (cross-sectional studies and longitudinal studies) and adolescent gender. For cross-sectional data, each dimension of the measure was treated as an independent effect size estimate. For longitudinal data, a summary effect size was calculated to present data over all time points to reinforce the credibility of findings. As to subgroup analysis by adolescent gender, we selected the studies including data collected from boys and girls separately and examined the effect size estimates for two groups.

### 2.3. Effect Size Computation 

Cohen’s *d* was used to compute the effect size for the standardized difference between means of perceived paternal and maternal attributes on a continuous measure. The effect size was calculated by subtracting the mean score for perceived maternal attributes from the mean score for perceived paternal attributes, divided by the within-groups’ standard deviation. Thus, for each independent estimate of effects, the effect size was computed as *d* = (M_p_ − M_m_)/S_w_ (M_p_ = the mean score for perceived paternal attributes; M_m_ = the mean score for perceived maternal attributes; S_w_ = the pooled within-groups standard deviation).

Positive values of *d* represent higher scores on perceived paternal parenting attributes, whereas negative values represent higher scores on perceived maternal parenting attributes. Cohen [78] provided the guidelines for effect size interpretation. Effect sizes of *d* = 0.20, 0.50, and 0.80 are interpreted as small, medium, and large effects, respectively. If statistical information necessary for the computation of effect sizes there was missing, the study was excluded from the meta-analysis. In comparison to small-scale studies, studies with larger sample size were given more weight to compute weighted and pooled effect sizes. The generic inverse variance method was used to determine weight in the analysis, which means that the weight given to each study equals the inverse of the variance of the effect estimate.

### 2.4. Variance Ratio Computation

Variance ratios (VR) were computed for each independent estimate of effects by dividing the variance of perceived paternal attributes by the variance of perceived maternal attributes (VR = Variance_p_/Variance_m_). Thus, a VR greater than 1 indicates greater variability of perceived paternal attributes, whereas a VR less than 1 indicates greater variability of perceived maternal attributes. The calculation of VRs was frequently used in previous studies [79,80,81]. To aggregate the VRs for the current meta-analysis, a base −10 log transform was performed to present the mean weighted antilog variance ratio [81,82].

### 2.5. Random-Effect Model 

The fixed-effect model and the random-effect model are two analysis models used in a meta-analysis with different assumptions of the nature of the studies. Under the fixed-effect model, the true effect size is assumed to be identical and the same in all studies [83]. In contrast, the random-effect model assumes the true effect size to vary across studies. The random-effect model does not discount a small-scale study by giving it a smaller weight or assign too much weight to a study with a large sample. The weights assigned to each study in the random-effect model fall in a relatively narrow and balanced range. Given that the included studies employed various types of measures and dimensions, we expected that the samples of effect sizes would be significantly heterogeneous across studies. Therefore, we adopted the random-effect model to calculate pooled effect sizes (95% confidence interval).

### 2.6. Between-Study Heterogeneity 

We used *I*^2^ to quantify the effect of heterogeneity, which judges the consistency across studies. It describes the percentage of total variation across studies that is due to heterogeneity rather than chance [84]. The formula is

*I*^2^ = 100% × (*Q* − *df*)/*Q* (1)

where *Q* is the chi-squared heterogeneity statistic and *df* is the degrees of freedom. The percentage of *I*^2^ lies between 0% and 100%, with a value of 0% indicates no observed heterogeneity. The value for *I*^2^ of 25%, 50%, and 75% can be interpreted as low, moderate, and high heterogeneity, respectively.

## 3. Results

### 3.1. Search Results

The original search resulted in 3271 articles. Three additional articles were identified by reviewing the reference lists of the retrieved articles. After excluding 635 duplicates, we imported the remaining articles into EndNote for titles and abstracts screening. Then 379 articles were selected for full-text examination. Given that most studies did not separately examine paternal and maternal parenting attributes based on adolescents’ perceptions, we eventually found 46 articles that provided relevant and potentially eligible information for coding. Among them, three articles were eliminated, because the data reported in these articles had already been included in other studies, which duplicated the findings [8,29,85]. The final sample of studies included data from 43 articles. A summary of the selection process is presented in Figure 1.

### 3.2. Study Characteristics

The characteristics of the 43 included studies are presented in Table 1. All the studies provided adequate information to derive measures of effect sizes (e.g., means and standard deviations for paternal and maternal parenting attributes). Among the included studies, the minimum sample size of participants was 120. A total of 55,759 adolescents were included in the meta-analysis. The adolescent participants had a mean age ranged from 11.92 to 17.39. As to the study design, 38 studies adopted the cross-sectional design and five studies were based on longitudinal design. Fifteen studies reported data for adolescent males and adolescent females separately. Concerning the background socio-demographic information of the participants, as the detailedness of the information and the criteria adopted (e.g., social class) varied across studies, readers are advised to refer to such information in the original studies.

Different measures of parenting attributes, including the original, modified or subscale versions, were used in the included studies. The measures used in the studies included the Parent Behaviors Measure [44,45,46], Inventory of Parent and Peer Attachment [22,47,48,49,50,51,52], Parental Bonding Instrument [53,54,55,56], Children’s Report of Parental Behavior Inventory [32,57,58], Parental Acceptance-Rejection Questionnaire [59,60,61], Egna Minnen Beträffande Uppfostran for Children [49,62,63], Perceptions of Parents Scale [64,65], Parent-Child Subsystem Quality Scale [27,28], Parental Monitoring Measure [58,62], Psychological Control Scale [58,62], Paternal/Maternal Psychological Control Scale [33,66], Child Rearing Practice Report [57], Father-/Mother-Adolescent Communication Scale [23], Paternal and Maternal Parenting Style Scale [21], Chinese Paternal and Maternal Control Scale [21], Authoritative Parenting Index [67], Closeness to Parents Scale [25], Inventory of Parental Influence [60], Paternal/Maternal Treatment Scale [20], Paternal/Maternal Parenting Style Scale [20], Parent-Adolescent Relationship Questionnaire [26], Ghent Parental Behavior Scale [62], Harsh Discipline Scale [68], Parents as Social Context Questionnaire [18], Autonomy-Control Scale [61], Disrespect Scale [69], and Family Adaptation and Cohesion Evaluation Scales II Inventory [70]. Other studies used self-developed measures [32,71,72,73,74]. Regarding the adaptation of measures among the included studies, thirty-one studies used translated measures [18,20,22,25,26,32,44,45,46,47,48,49,50,51,52,53,54,55,56,58,59,60,61,63,64,65,67,68,69,70,75], ten used non-translated measures [21,23,28,29,33,66,71,72,73,74], and two of them used both translated and non-translated measures [32,62]. The psychometric properties of the measures, such as validity and reliability, can be retrieved in each study included in this meta-analysis. 

In addition, some studies examined several dimensions of parenting attributes for the same sample (Table 1). Therefore, a total of 33 dimensions of parenting attributes by fathers and mothers were included in the analyses. These dimensions consisted of 19 positive parenting attributes and 14 negative parenting attributes. The positive attributes were comprised of support, reasoning, monitoring, autonomy, trust, communication, warmth, responsiveness, demandingness, control, care, involvement, parent–child relationships, behavioral control, concern, behavioral guidance, emotional warmth, cohesion, and the composite score of the Inventory of Parent and Peer Attachment measure (i.e., trust, communication, and alienation). The negative parenting attributes covered punitiveness, love withdrawal, alienation, indulgence, rejection, psychological control, overprotection, anxious rearing, indifference, pressure, harshness, harsh discipline, guilt induction, and permissiveness.

### 3.3. Overall Perceptions of Paternal and Maternal Parenting Attributes

Forty-three studies with 99 computed effect sizes contributed to the overall effect analysis (Table 2). Noteworthily, cross-sectional data and Wave 1 data of longitudinal studies were included. Data with multiple outcomes were all treated as independent estimates of effects. Among all the independent effect size estimates, 61 dimension outcomes revealed higher perceived maternal parenting attributes and 38 dimension outcomes showed higher perceived paternal parenting attributes.

For the principal outcome, the weighted and pooled effect size for the overall perceptions of parenting attributes among Chinese adolescents was −0.12 (95% CI: −0.17 to −0.06; *p* < 0.001), showing that perceived maternal parenting attributes were significantly more positive than perceived paternal parenting attributes. Therefore, Hypothesis 1 was supported. There was evidence of substantial heterogeneity of the combined effect size (*Q* = 4066.63, *p* < 0.001; *I*^2^ = 97.6%). Mean weighted antilog variance ratio of the overall effect was 1.021, which indicated a slightly 2.1% greater variability in perceived paternal parenting attributes than maternal parenting attributes.

### 3.4. Subgroup Analysis—Study Design

We performed meta-analyses for cross-sectional and longitudinal studies separately (Table 2). Thirty-eight studies with 90 computed effect sizes contributed to the cross-sectional analysis. For the four longitudinal studies, effect size estimates at all time points of each study were aggregated to compute a summary effect size, which resulted in 9 computed effect sizes. For instance, results of a six-year longitudinal study were included in two publications by Shek and colleagues [27,29], with one publication reported data from Wave 1 to Wave 3 and another contained data from Wave 4 to Wave 6. All data collected from Wave 1 to Wave 6 were aggregated to compute a summary effect in the longitudinal data analysis.

As a result, the effect size for cross-sectional data and longitudinal data were −0.10 (95% CI: −0.16 to −0.04; *p* < 0.001) and −0.26 (95% CI: −0.49 to −0.02; *p* < 0.05), respectively. The outcome suggested that Chinese adolescents in both study designs perceived higher maternal parenting attributes than perceived paternal parenting attributes. Thus, the results did not support Hypothesis 2. No significant difference was observed on the weighted effect sizes between cross-sectional and longitudinal data (*p* > 0.05). Mean weighted antilog variance ratios of the cross-sectional data and longitudinal data were 1.021 and 1.012, respectively. Both types of study design had slightly greater variability in the perceptions of paternal parenting attributes than maternal parenting attributes. In addition, there was evidence of substantial heterogeneity for the pooled effect size for the pooled effect size for both types of study design (Cross-sectional data: *Q* = 3174.73, *p* < 0.001; *I*^2^ = 97.2%; Longitudinal data: *Q* = 3383.05, *p* < 0.001; *I*^2^ = 99.8%).

### 3.5. Subgroup Analysis—Gender

A total of 14 studies reported sufficient data separately on adolescent males and adolescent females (Table 2). Thirty computed effect sizes were calculated for each gender. Noteworthily, Kwok and Shek’s research [23] was excluded in the gender subgroup analyses due to insufficient information to calculate the gender proportion in the study.

The effect sizes for adolescent males and adolescent females were −0.07 (95% CI: −0.15 to 0.01; *p* = 0.07) and −0.10 (95% CI: −0.19 to −0.00; *p* < 0.05), respectively. The outcomes suggested that only Chinese adolescent females perceived significantly higher maternal parenting attributes than they perceived paternal parenting attributes. Hypothesis 3 was supported. No significant difference was observed on the weighted effect sizes between adolescent males and adolescent females (*p* > 0.05). Mean weighted antilog variance ratios of the adolescent males and adolescent females were 1.012 and 1.009, respectively. The two variance ratios indicated a close to gender similarities in variability, with 1.2% and 0.9% greater variability in the perceptions of paternal parenting attributes than maternal parenting attributes. In addition, there was evidence of substantial heterogeneity for the pooled effect size for both genders (adolescent males: *Q* = 396.51, *p* < 0.001; *I*^2^ = 92.7%; adolescent females: *Q* = 645.59, *p* < 0.001; *I*^2^ = 95.5%).

In summary, several major observations emerged from the meta-analysis. First, overall speaking, perceived maternal parenting attributes were significantly more positive than did paternal parenting attributes (*d* = −0.12; *p* < 0.001). Second, the variability of perceived paternal parenting attributes was slightly greater than maternal parenting attributes (VR = 1.021). Third, perceived maternal parenting attributes were significantly more positive than did perceived paternal parenting attributes in both cross-sectional (*d* = −0.10; *p* < 0.001) and longitudinal studies (*d* = −0.26; *p* < 0.05). Finally, based on studies providing both male and female data, only adolescent females perceived maternal parenting attributes significantly more positive than paternal parenting attributes (*d* = −0.10; *p* < 0.05).

## 4. Discussion

This meta-analysis study examined perceived parental differences between Chinese mothers and fathers based on 43 studies. There are some unique features in the study. First, as a response to the call for more research on parenting in Asian cultures [10,86], this meta-analysis study examined perceived parental differences in Chinese contexts. Second, this study attempts to clarify equivocal findings on parental differences by examining both positive and negative parenting attributes. Third, we conducted subgroup analyses to explore whether adolescent gender and study design could explain parental differences between Chinese mothers and fathers.

Results showed that Chinese mothers’ parenting attributes were generally perceived to be more positive than those of fathers. Our results are in line with previous findings, suggesting that Chinese mothers take dominant parenting roles and are more caring, responsive, and supportive than fathers [10,87,88]. Despite the increasing emphasis on paternal involvement in Western countries, research has shown that mothers still play a significant role in day-to-day parenting in Chinese contexts. More interestingly, results indicated that Chinese adolescents possessed more positive perceptions of maternal parenting, although some studies revealed mothers used more negative maternal parenting attributes than did fathers. There are some possible contextual explanations. First, some scholars argued that Chinese parents’ use of control is often to foster family cohesion rather than express hostility or revoke conflicts [89]. Besides, As Chua [90] described in her book about tiger mothers in contemporary Chinese societies, many Chinese parents believe that “the best way to protect their children is by preparing them for the future, letting them see what they’re capable of, and arming them with skills, work habits, and inner confidence that no one can ever take away.” They want their children to succeed in examinations, and thus gain access to well-reputed schools and well-paid jobs in the future. Therefore, Chinese adolescents may perceive maternal parenting as “tough love”, which reflects mothers’ intention to help their children reach the highest potentials and achievements [7]. As Leung and Shek [88] suggested, Chinese mothers showed a higher level of sacrifice for their children’s education than did fathers. If maternal sacrifice is appreciated by children, it may mitigate adolescents’ negative perceptions of certain parenting attributes, such as harsh discipline and overprotection. In Bach and Christensen’s study [91] conducted in Singapore, one interviewee considered her mother a good mother, because she sacrificed a lot to push her children to achieve academic and work success. Therefore, if adolescents appreciate their mothers’ sacrifice and agree to the underlying rationale of harsh parenting behaviors, they may perceive more positive maternal parenting attributes.

Our result indicated that the variability of perceived paternal parenting attributes was slightly greater than maternal parenting attributes. This result suggests that Chinese mothers’ parenting behaviors show a relatively consistent pattern, while paternal parenting likely varies across families. Chinese fathers are traditionally expected to take financial responsibilities for the family, and thus may have limited time and opportunities to interact with their children. Their involvement in parenting may be profoundly constrained by varying developmental history and external working conditions. Besides, as fathers are still regarded as the lead of Chinese families, fathers’ involvement, such as emotional warmth, can be more important to adolescents than mothers’ day-to-day parenting [63]. Therefore, the variation in perceived paternal parenting may be related to adolescents’ higher sensitivity to fathers’ involvement in their lives. Another possible explanation concerns the increasing involvement of fathers in urban cities in China. On the one hand, urban families may resonate with the increasing emphasis on father involvement in childcare in contemporary Western societies. On the other hand, the movement towards greater gender equality and higher female employment rate in urban areas encourage fathers to shoulder more caregiving responsibilities than before.

Subgroup analysis based on study design revealed that the positive perceptions of maternal parenting were relatively stable concurrently and longitudinally. This result is in line with previous research. As revealed in Shek and Dou’s study [37], although both maternal and paternal parenting attributes decreased over time, maternal attributes were stronger than paternal attributes across the six years of secondary education. This observation suggests perceived parental differences are relatively stable throughout the adolescent years. However, as longitudinal studies are rare in different Chinese contexts, there is a need to replicate the present findings.

Based on 14 studies, subgroup analysis based on adolescent gender indicated that only girls perceived maternal parenting attributes to be more positive than those of fathers. This result also echoes earlier findings, which suggest that girls generally perceive relatively higher levels of maternal support and control than boys during adolescence [92]. Besides, existing research suggests that adolescent boys tend to be less obedient toward parents and show a higher rate of achievement autonomy than do girls [93]. Shek and Dou’s study [37] revealed a slower decrease in parental behavioral control over adolescent girls than boys. Chinese parents may gradually reduce their control and support to boys entering adolescence, which leads to differentiated perceptions of parental behaviors among boys and girls. However, as only 14 studies were included in subgroup analysis, the findings should be interpreted with caution.

This meta-analysis study contributes to the field by revealing perceived parental differences in Chinese cultures. Results should be extrapolated to other cultures with caution. Our findings follow the patterns commonly observed across cultures that mothers are perceived to be more caring and responsible than fathers. However, it is noteworthy that some idiosyncratic characteristics of the Chinese context may influence adolescents’ perceptions. For example, Chinese mothers have traditionally been expected to be dutiful wives and loving mothers and thus may experience more substantial role strain in parenting. Besides, parents with only one child may have great ambitions for their child and thus be more caring but also controlling. These factors should be considered when interpreting the findings.

The present study has some limitations. First, the study examined parental differences based on the perceptions of adolescents. It would be illuminating to collect parental data reported by fathers and mothers [21]. Second, as this study focused on parental differences per se, we cannot identify the impact of parental differences on adolescent development. Third, this study only included research in English databases. A future study can include research indexed in Chinese databases to provide additional evidence and prevent publication bias [94]. Fourth, we only conducted subgroup analyses based on gender and study design. It would be interesting to analyze whether perceived parenting attributes differ among adolescent populations with different characteristics, such as geographic location, socioeconomic background, and family structure, in future studies. However, such analyses may be difficult, because the detailedness of the information and criteria involved (e.g., income brackets) varied across studies. There is a need to standardize to allow meaningful comparisons.

## 5. Conclusions

Despite the above limitations, this meta-analysis study adds value to the existing literature by examining parental differences between mothers and fathers in Chinese contexts. The present study is a constructive response to the observation that studies on family quality of life are inadequate in different Chinese studies [85,95]. Results demonstrated that perceived parenting attributes of Chinese mothers were generally more positive than those of Chinese fathers in both cross-sectional and longitudinal studies. Besides, the variability of paternal parenting attributes was greater than that of maternal parenting attributes. Moreover, subgroup analysis showed that only adolescent girls perceived maternal parenting attributes to be more positive than those of fathers. This study offers insight into the family environment and functioning for Chinese adolescents. Earlier research has revealed a relatively low level of paternal involvement but an indispensable role of fathers in promoting adolescent development [35]. As our findings revealed less positive perceptions of paternal parenting attributes, Chinese fathers should be more aware of their parenting responsibilities and actively provide more support and warmth to their children. Moreover, high levels of maternal involvement in parenting suggest that the traditional work distribution of household and parenting tasks still prevails. The findings also suggest that Chinese mothers may experience more substantial role strain in the family process. There is a need to reflect on the pre-set cultural beliefs in Chinese families and the ideologies of gender roles of parenting on Chinese mothers.

## Figures and Tables

**Table 1:** Characteristics of included articles in the meta-analysis.

Study	Geographic Location	*N*	Mean Age	Female %	SD	M/F	Measure	Dimension
Wang, M. (2019) [18]	Jinan	867	12.47	46.4	CS	N	Parental Warmth subscale of Parents as Social Context Questionnaire (PASCQ)	Warmth
Shek (2000) [20]	Hong Kong	429 (T1)	12–16	49.3	LS	Y	Paternal/Maternal Treatment Scale (PTS; MTS)	Concern; Harshness
		378 (T2)					Paternal/Maternal Parenting Style Scale (PPS; MPS)	Responsiveness; Demandingness
Leung and Shek (2014) [21]	Hong Kong	275	13.56	51.3	CS	N	Paternal and Maternal Parenting Style Scale (PPS; MPS)	Responsiveness; Demandingness
							Chinese Paternal and Maternal Control Scale (APCS; AMCS)	Control
Chen, B. (2017) [22]	Shanghai	384	15.13	50.4	CS	N	Inventory of Parent and Peer Attachment (IPPA)	Trust; Communication; Alienation
Lai Kwok and Shek (2010) [23]	Hong Kong	5557	13.87	NR	CS	Y	Father-/Mother-Adolescent Communication Scale (FACS; MACS)	Communication
Liu, Q., Fang et al. (2013) [25]	Jinan and Beijing	4559	15.00	51.5	CS	Y	Closeness to Parents Scale	Parent-child Relationship
Su, Yu et al. (2018) [26]	Guangzhou	1490 (T1–T3)	12.03	45.4	LS	N	Parent-child Relationship Questionnaire	Parent-child Relationship
Shek, Zhu, & Ma (2018) [27]; Shek, Zhu, & Dou (2019) [28]	Hong Kong	3328 (T1)	12.59–15.57	47.6–48.7	LS	N	Parent-Child Subsystem Quality Scale (PCSQS)	Psychological Control; Behavioral Control; Parent-child Relationship
		2905 (T2)						
		2860 (T3)						
		3074 (T4–T6)						
Chen, X., Liu, & Li (2000) [32]	Shanghai	202	11.92	52.5	CS	Y	Children’s Report of Parental Behavior Inventory (CRPBI)	Warmth; Harshness
							Self-developed 6-item Measure	Indulgence
Shek (2007) [33]	Hong Kong	3017 (T1)	12.65	55.7	LS	Y	Paternal/Maternal Psychological Control Scale (CPPCS; CMPCS) – Chinese version	Psychological Control
		2758 (T2)						
Bush, Peterson et al. (2002) [44]	Beijing	480	15.42	50.4	CS	N	Parent Behavior Measure (PBM)	Support; Reasoning; Monitoring; Punitiveness; Love Withdrawal; Autonomy
Peterson, Cobas et al. (2005) [45]	Beijing	497	15.42	50.4	CS	N	Parent Behavior Measure (PBM)	Support; Reasoning; Monitoring; Punitiveness; Autonomy
Xia, Wang et al. (2015) [46]	Hangzhou	589	15.76	60.0	CS	N	Parent Behavior Measure (PBM)—Modified	Guilt Induction; Reasoning; Monitoring; Punitiveness; Love Withdrawal; Involvement; Permissiveness
Jiang, You et al. (2017) [47]	Qingyuan	658	13.58	40.1	CS	N	Inventory of Parent and Peer Attachment (IPPA)	Trust; Communication; Alienation
Li, J., Delvecchio et al. (2014) [48]	Guangzhou	350	14.17	47.7	CS	N	Inventory of Parent and Peer Attachment (IPPA)	Trust; Communication; Alienation
Mothander and Wang (2014) [49]	Beijing	510	14.05–17.14	52.9	CS	Y	Egna Minnen Beträffande Uppfostran for Children (EMBU-C)	Overprotection; Rejection; Warmth; Anxious Rearing
							Inventory of Parent and Peer Attachment (IPPA)	Composite Score (Trust, Communication, Alienation)
Nie, Li et al. (2016) [50]	Guangzhou	607	14.43	52.9	CS	N	Inventory of Parent and Peer Attachment (IPPA)	Composite Score (Trust, Communication, Alienation)
Pan, Zhang et al. (2016) [51]	Six regions of China	1506	15.21	49.8	CS	Y	Inventory of Parent and Peer Attachment (IPPA)	Composite Score (Trust, Communication, Alienation)
Song, Thompson, & Ferrer (2009) [52]	Guangzhou	314	13.76–16.72	54.5	CS	N	Inventory of Parent and Peer Attachment (IPPA)	Trust; Communication; Alienation
Lai and McBride-Chang (2001) [53]	Hong Kong	120	16.10	62.5	CS	N	Parental Bonding Instrument (PBI)	Care; Overprotection
Yu, Wang et al. (2007) [54]	China	167	15.90	55.7	CS	N	Parental Bonding Instrument (PBI)	Care; Overprotection; Autonomy
Zhu, Luo et al. (2014) [55]	Changsha	594	16.70	56.6	CS	N	Parental Bonding Instrument (PBI)	Overprotection
Ngai, Cheung et al. (2018) [56]	Hong Kong	1997	14.60	50.3	CS	Y	Parental Bonding Instrument (PBI)—Modified	Care; Indifference; Autonomy; Overprotection
Chen, X., Bian et al. (2010) [57]	Miyun District	1359	13.60–13.90	47.8	CS	N	Children’s Report of Parental Behavior Inventory (CRPBI) & Child Rearing Practices Report (CRPR)—Modified	Warmth; Harshness; Autonomy
Stolz, Barber et al. (2004) [58]	Beijing	970	14–17	54.5	CS	N	Acceptance subscale of Children’s Report of Parental Behavior Inventory (CRPBI)	Support
							Parental Monitoring Measure	Monitoring
							Psychological Control Scale	Psychological Control
Li, X. (2014) [59]	Southeast China	305	13.57	55.0	CS	Y	Parental Acceptance-Rejection Questionnaire (PARQ)	Rejection
Quach, Epstein et al. (2015) [60]	Beijing	997	16.60 –17.10	52.0	CS	Y	Warmth/Affection Scale of Parental Acceptance Rejection Questionnaire (PARQ)	Warmth
							Inventory of Parental Influence (IPI)	Pressure
Wong, De Man, & Leung (2002) [61]	Hong Kong	365	14.09	51.2	CS	N	Warmth/Affection subscale of Parental Acceptance-Rejection Questionnaire (PARQ)	Warmth
							Adolescent subscale of Autonomy-Control Scale	Autonomy
Wang, J., Shi et al. (2019) [62]	Beijing	2775	15.70	55.3	CS	N	Modified from Egna Minnen Beträffande Uppfostran for Children (EMBU-C), Ghent Parental Behavior Scale (GPBS), Psychological Control Scale, & Parental Monitoring Measure	Warmth; Behavioral Guidance; Harsh Discipline
Xu, J., Ni et al. (2017) [63]	Guangdong	1345	13.70	40.7	CS	Y	Egna Minnen Beträffande Uppfostran for Children	Overprotection; Rejection; Emotional Warmth
To, Helwig, & Yang (2017) [64]	Guangzhou and Northern Guangdong	395	13.90–16.99	52.7	CS	N	Perception of Parents Scale (POPS)	Autonomy; Responsiveness
Liu, G., Zhang et al. (2013) [65]	Jinan	550	13.58–17.39	54.9	CS	N	Perceptions of Parents Scale (POPS)	Involvement
Liu and Chang (2016) [66]	Northern Taiwan	329	13.32	42.9	CS	N	Paternal/Maternal Psychological Control Scale (PPCS; MPCS)	Psychological Control
							Paternal/Maternal Behavioral Control Scale (PBCS; MBCS)	Behavioral Control
Li, X. M., Mao et al. (2010) [67]	Nanjing	982	15.13–15.99	49.6	CS	Y	Authoritative Parenting Index	Responsiveness; Demandingness
Wang, M., Deng, & Du. (2018) [68]	Eastern China	815	12.55	42.7	CS	Y	Harsh Discipline Scale	Harsh Discipline
Xu, X., Lou et al. (2017) [69]	Guangdong, Hubei, and Henan	401	16.13	60.6	CS	N	Psychological Control – Disrespect Scale (PCDS)	Psychological Control
Zhao, Liu, & Wang (2015) [70]	Henan	241	13.86–13.92	41.9	CS	N	Family Adaptation and Cohesion Evaluation Scales II Inventory (FACES)	Cohesion
Chen and Astor (2011) [71]	Taiwan	7841	Grade 10–12	51.3	CS	Y	Self-developed 5-item Measure	Monitoring
Chen and Astor (2012) [72]	Taiwan	3058	Grade 7–9	49.5	CS	Y	Self-developed 5-item Measure	Monitoring
Liu, R., Lin, & Chen (2010) [73]	Fuzhou	1924	12–13	49.0	CS	N	Self-developed 7-item Measure	Responsiveness
Wang, Y., Chan, Lin et al. (2015) [74]	Taiwan	1990	13.30	50.3	CS	N	Self-developed 8-item Measure	Warmth; Harsh Discipline
Pan, Hu et al. (2017) [75]	Sichuan and Chongqing	595	12.86	52.4	CS	N	Inventory of Parent and Peer Attachment (IPPA)	Composite Score (Trust, Communication, Alienation)

Note. SD: Study design; CS: Cross-sectional data; LS: Longitudinal data; M/F: Reported separately in adolescent males and adolescent females; Y: Yes; N: No; NR: Not reported.

**Table 2:** Number of Studies, Number of Effect Sizes, Mean Weighted Antilog Variance Ratio, Mean Weighted Effect Sizes, 95% Confidence Intervals, and Heterogeneity Statistic for Perceived Parental Differences in Parenting Behaviors.

Variable	*k*	#ES	VR	*d*	95% CI		Heterogeneity Test	
					Lower Limit	Upper Limit	Q-Value	*I* ^2^
Overall Effect ^a^	42	99	1.021	−0.12 ***	−0.17	−0.06	4066.63 ***	97.6%
Study Design								
Cross-sectional	38	90	1.021	−0.10 ***	−0.16	−0.04	3174.73 ***	97.2%
Longitudinal ^b^	4	9	1.012	−0.26 *	−0.49	−0.02	3383.05 ***	99.8%
Gender								
Adolescent males ^a^	14	30	1.012	−0.07	−0.15	0.01	396.51 ***	92.7%
Adolescent females ^a^	14	30	1.009	−0.10 *	−0.19	−0.00	645.59 ***	95.5%

Note. *k* = number of independent studies; #ES = number of effect sizes; VR = mean weighted antilog variance ratio (greater than 1 indicates greater variability in the perceptions of paternal parenting attributes); *d* = mean weighted effect size; CI = confidence interval of the mean weighted effect size. Negative value of effect size indicates higher level of perceived maternal parenting attributes than perceived paternal parenting attributes. *I*^2^ = percentage of variance that is attributable to heterogeneity. ^a^ Only Wave 1 data in longitudinal studies were included in the analysis. ^b^ A summary effect measure of all waves was computed and included in the analysis. *** *p* < 0.001; * *p* < 0.05.

## References

[1] Brown L., Iyengar S. (2008). Parenting styles: The impact on student achievement. Marriage Fam. Rev..

[2] Whittle S., Simmons J.G., Dennison M., Vijayakumar N., Schwartz O., Yap M.B.H., Sheeber L., Allen N.B. (2014). Positive parenting predicts the development of adolescent brain structure: A longitudinal study. Dev. Cogn. Neurosci..

[3] Cui M., Conger R.D., Bryant C.M., Elder G.H. (2002). Parental behavior and the quality of adolescent friendships: A social-contextual perspective. J. Marriage Fam..

[4] Collins W.A., Russell G. (1991). Mother-child and father-child relationships in middle childhood and adolescence: A developmental analysis. Dev. Rev..

[5] Chen J.J., Sun P., Yu Z. (2017). A comparative study on parenting of preschool children between the Chinese in China and Chinese immigrants in the United States. J. Fam. Issues.

[6] Leung J.T.Y., Busiol D. (2016). Adolescents growing up in a “greenhouse:” A literature review. Int. J. Child Adolesc. Health.

[7] Katz C. (2018). The angel of geography: Superman, tiger mother, aspiration management, and the child as waste. Prog. Hum. Geogr..

[8] Leung J.T.Y., Shek D.T.L. (2016). The influence of parental beliefs on the development of Chinese adolescents experiencing economic disadvantage: Maternal control as a mediator. J. Fam. Issues.

[9] Shek D.T.L. (1998). Adolescents’ perceptions of paternal and maternal parenting styles in a Chinese context. J. Psychol..

[10] Shek D.T.L. (2008). Perceived parental control and parent-child relational qualities in early adolescents in Hong Kong: Parent gender, child gender and grade differences. Sex Roles.

[11] Bem S.L. (1974). The measurement of psychological androgyny. J. Consult. Clin. Psychol..

[12] Klimes-Dougan B., Brand A.E., Zahn-Waxler C., Usher B., Hastings P.D., Kendziora K., Garside R.B. (2007). Parental emotion socialization in adolescence: Differences in sex, age and problem status. Soc. Dev..

[13] Van Zalk N., Tillfors M., Trost K. (2018). Mothers’ and fathers’ worry and over-control: One step closer to understanding early adolescent social anxiety. Child Psychiatry Hum. Dev..

[14] Mastrotheodoros S., Van der Graaff J., Deković M., Meeus W.H.J., Branje S.J.T. (2018). Coming closer in adolescence: Convergence in mother, father, and adolescent reports of parenting. J. Res. Adolesc..

[15] Shek D.T.L., Sun R.C.F., Selin H. (2014). Parenting in Hong Kong: Traditional Chinese cultural roots and contemporary phenomena. Parenting Across Cultures: Childrearing, Motherhood and Fatherhood in Non-Western Cultures.

[16] Maccoby E.E., Martin J.A., Mussen P.H. (1983). Socialization in the context of the family: Parent–child interaction. Handbook of Child Psychology: Socialization, Personality and Social Development.

[17] Baumrind D. (1991). The influence of parenting style on adolescent competence and substance use. J. Early Adolesc..

[18] Wang M. (2019). Harsh parenting and adolescent aggression: Adolescents’ effortful control as the mediator and parental warmth as the moderator. Child Abuse Negl..

[19] Deater-Deckard K., Lansford J.E., Malone P.S., Alampay L.P., Sorbring E., Bacchini D., Bombi A.S., Bornstein M.H., Chang L., Di Giunta L. (2011). The association between parental warmth and control in thirteen cultural groups. J. Fam. Psychol..

[20] Shek D.T.L. (2000). Differences between fathers and mothers in the treatment of, and relationship with, their teenage children: Perceptions of Chinese adolescents. Adolescence.

[21] Leung J.T.Y., Shek D.T.L. (2014). Parent-adolescent discrepancies in perceived parenting characteristics and adolescent developmental outcomes in poor Chinese families. J. Child Fam. Stud..

[22] Chen B.B. (2017). Parent–adolescent attachment and procrastination: The mediating role of self-worth. J. Genet. Psychol..

[23] Lai Kwok S.Y.C., Shek D.T.L. (2010). Hopelessness, parent-adolescent communication, and suicidal ideation among Chinese adolescents in Hong Kong. Suicide Life Threat. Behav..

[24] Shek D.T.L., Lee B.M., Lee T.Y., Lam C.M. (2006). Frequency, satisfaction and quality dimensions of perceived parent-adolescent communication among Chinese adolescents in Hong Kong. Int. J. Adolesc. Med. Health.

[25] Liu Q.X., Fang X.Y., Zhou Z.K., Zhang J.T., Deng L.Y. (2013). Perceived parent-adolescent relationship, perceived parental online behaviors and pathological Internet use among adolescents: Gender-specific differences. PLoS ONE.

[26] Su B., Yu C., Zhang W., Su Q., Zhu J., Jiang Y. (2018). Father-child longitudinal relationship: Parental monitoring and internet gaming disorder in Chinese adolescents. Front. Psychol..

[27] Shek D.T.L., Zhu X., Ma C.M.S. (2018). The influence of parental control and parent-child relational qualities on adolescent internet addiction: A 3-year longitudinal study in Hong Kong. Front. Psychol..

[28] Shek D.T.L., Zhu X., Dou D. (2019). Influence of family processes on Internet addiction among late adolescents in Hong Kong. Front. Psychiatry.

[29] Shek D.T.L., Zhu X. (2019). Paternal and maternal influence on delinquency among early adolescents in Hong Kong. Int. J. Environ. Res. Public Health.

[30] Hoeve M., Dubas J.S., Eichelsheim V.I., van der Laan P.H., Smeenk W., Gerris J.R.M. (2009). The relationship between parenting and delinquency: A meta-analysis. J. Abnorm. Child Psychol..

[31] Shek D.T.L. (1995). Chinese adolescents’ perceptions of parenting styles of fathers and mothers. J. Genet. Psychol..

[32] Chen X., Liu M., Li D. (2000). Parental warmth, control, and indulgence and their relations to adjustment in Chinese children: A longitudinal study. J. Fam. Psychol..

[33] Shek D.T.L. (2007). A longitudinal study of perceived parental psychological control and psychological well-being in Chinese adolescents in Hong Kong. J. Clin. Psychol..

[34] Guo Q., Feng L. (2017). The associations between perceived parenting styles, empathy, and altruistic choices in economic games: A study of Chinese children. Front. Psychol..

[35] Shek D.T.L. (2005). Perceived parental control and parent–child relational qualities in Chinese adolescents in Hong Kong. Sex Roles.

[36] Adachi P., Willoughby T. (2015). Interpreting effect sizes when controlling for stability effects in longitudinal autoregressive models: Implications for psychological science. Eur. J. Dev. Psychol..

[37] Shek D.T.L., Dou D. (2020). Perceived parenting and parent-child relational qualities in fathers and mothers: Longitudinal findings based on Hong Kong adolescents. Int. J. Environ. Res. Public Health.

[38] Seiffge-Krenke I., Pakalniskiene V. (2011). Who shapes whom in the family: Reciprocal links between autonomy support in the family and parents’ and adolescents’ coping behaviors. J. Youth Adolesc..

[39] Smetana J.G., Daddis C. (2002). Domain-specific antecedents of parental psychological control and monitoring: The role of parenting beliefs and practices. Child Dev..

[40] Bussey K., Bandura A. (1999). Social cognitive theory of gender development and differentiation. Psychol. Rev..

[41] Branje S.J.T., Laursen B., Collins W.A., Vangelisti A.L. (2013). Parent-child communication during adolescence. Handbook of Family Communication.

[42] Barber B.K., Stolz H.E., Olsen J.A., Collins W.A., Burchinal M. (2005). Parental support, psychological control, and behavioral control: Assessing relevance across time, culture, and method. Monogr. Soc. Res. Child Dev..

[43] Dhand N.K., Khatkar M.S. Statulator: An Online Statistical Calculator. Sample Size Calculator for Comparing Two Paired Means. http://statulator.com/SampleSize/ss2PM.html.

[44] Bush K.R., Peterson G.W., Cobas J.A., Supple A.J. (2002). Adolescents’ perceptions of parental behaviors as predictors of adolescent self-esteem in mainland China. Sociol. Inq..

[45] Peterson G.W., Cobas J.A., Bush K.R., Supple A., Wilson S.M. (2005). Parent-youth relationships and the self-esteem of Chinese adolescents: Collectivism versus individualism. Marriage Fam. Rev..

[46] Xia Y.R., Wang C., Li W., Wilson S., Bush K.R., Peterson G. (2015). Chinese parenting behaviors, adolescent school adjustment, and problem behavior. Marriage Fam. Rev..

[47] Jiang Y., You J., Zheng X., Lin M.P. (2017). The qualities of attachment with significant others and self-compassion protect adolescents from non suicidal self-injury. Sch. Psychol. Q..

[48] Li J.B., Delvecchio E., Miconi D., Salcuni S., Di Riso D. (2014). Parental attachment among Chinese, Italian, and Costa Rican adolescents: A cross-cultural study. Pers. Individ. Dif..

[49] Mothander P.R., Wang M. (2014). Parental rearing, attachment, and social anxiety in Chinese adolescents. Youth Soc..

[50] Nie Y.G., Li J.B., Vazsonyi A.T. (2016). Self-control mediates the associations between parental attachment and prosocial behavior among Chinese adolescents. Pers. Individ. Dif..

[51] Pan Y., Zhang D., Liu Y., Ran G., Teng Z. (2016). Different effects of paternal and maternal attachment on psychological health among Chinese secondary school students. J. Child Fam. Stud..

[52] Song H., Thompson R.A., Ferrer E. (2009). Attachment and self-evaluation in Chinese adolescents: Age and gender differences. J. Adolesc..

[53] Lai K.W., McBride-Chang C. (2001). Suicidal ideation, parenting style, and family climate among Hong Kong adolescents. Int. J. Psychol..

[54] Yu R., Wang Z., Qian F., Jang K.L., Livesley W.J., Paris J., Mowei S., Wei W. (2007). Perceived parenting styles and disordered personality traits in adolescent and adult students and in personality disorder patients. Soc. Behav. Pers..

[55] Zhu H., Luo X., Cai T., Li Z., Liu W. (2014). Self-control and parental control mediate the relationship between negative emotions and emotional eating among adolescents. Appetite.

[56] Ngai S.S.Y., Cheung C.K., Xie L., Ng Y.H., Ngai H.L., Liu Y., Ho J.C. (2018). Psychometric properties of the Parental Bonding Instrument: Data from a Chinese adolescent sample in Hong Kong. J. Child Fam. Stud..

[57] Chen X., Bian Y., Xin T., Wang L., Silbereisen R.K. (2010). Perceived social change and childrearing attitudes in China. Eur. Psychol..

[58] Stolz H.E., Barber B.K., Olsen J.A., Erickson L.D., Bradford K.P., Maughan S.L., Ward D. (2004). Family and school socialization and adolescent academic achievement. Marriage Fam. Rev..

[59] Li X. (2014). Parental power–prestige and the effects of paternal versus maternal acceptance on the psychological adjustment of Chinese adolescents. Cross Cult. Res..

[60] Quach A.S., Epstein N.B., Riley P.J., Falconier M.K., Fang X. (2015). Effects of parental warmth and academic pressure on anxiety and depression symptoms in Chinese adolescents. J. Child Fam. Stud..

[61] Wong I.N., De Man A.F., Leung P.W.L. (2002). Perceived parental child rearing and suicidal ideation in Chinese adolescents. Soc. Behav. Pers..

[62] Wang J., Shi X., Yang Y., Zou H., Zhang W., Xu Q. (2019). The joint effect of paternal and maternal parenting behaviors on school engagement among Chinese adolescents: The mediating role of mastery goal. Front. Psychol..

[63] Xu J., Ni S., Ran M., Zhang C. (2017). The relationship between parenting styles and adolescents’ social anxiety in migrant families: A study in Guangdong, China. Front. Psychol..

[64] To S., Helwig C.C., Yang S. (2017). Predictors of children’s rights attitudes and psychological well-being among rural and urban mainland Chinese adolescents. Soc. Dev..

[65] Liu G., Zhang S., Zhang J., Lee C., Wang Y., Brownell M. (2013). Autonomous motivation and Chinese adolescents’ creative thinking: The moderating role of parental involvement. Creat. Res. J..

[66] Liu Y.L., Chang H.T. (2016). The role of effortful control in the relationships among parental control, intentional self-regulation, and adolescent obedience. J. Child Fam. Stud..

[67] Li X., Mao R., Stanton B., Zhao Q. (2010). Parental, behavioral, and psychological factors associated with cigarette smoking among secondary school students in Nanjing, China. J. Child Fam. Stud..

[68] Wang M., Deng X., Du X. (2018). Harsh parenting and academic achievement in Chinese adolescents: Potential mediating roles of effortful control and classroom engagement. J. Sch. Psychol..

[69] Xu X., Lou L., Wang L., Pang W. (2017). Adolescents’ perceived parental psychological control and test anxiety: Mediating role of academic self-efficacy. Soc. Behav. Pers..

[70] Zhao J., Liu X., Wang M. (2015). Parent-child cohesion, friend companionship and left-behind children’s emotional adaptation in rural China. Child Abuse Negl..

[71] Chen J.K., Astor R.A. (2011). School engagement, risky peers, and student-teacher relationships as mediators of school violence in Taiwanese vocational versus academically oriented high schools. J. Community Psychol..

[72] Chen J.K., Astor R.A. (2012). School variables as mediators of personal and family factors on school violence in Taiwanese junior high schools. Youth Soc..

[73] Liu R.X., Lin W., Chen Z. (2010). The effect of parental responsiveness on differences in psychological distress and delinquency between singleton and non-singleton Chinese adolescents. J. Child Fam. Stud..

[74] Wang L.Y.C., Chan H.Y., Lin C.W., Li J.R. (2015). Association of parental warmth and harsh discipline with developmental trajectories of depressive symptoms among adolescents in Chinese society. J. Fam. Psychol..

[75] Pan Y., Hu Y., Zhang D., Ran G., Li B., Liu C., Liu G., Luo S., Chen W. (2017). Parental and peer attachment and adolescents’ behaviors: The mediating role of psychological suzhi in a longitudinal study. Child. Youth Serv. Rev..

[76] The Cochrane Collaboration (2014). Review Manager (RevMan).

[77] Higgins J.P.T., Green S. Cochrane Handbook for Systematic Reviews of Interventions Version 5.1.0 (updated March 2011). https://handbook-5-1.cochrane.org/front_page.htm.

[78] Cohen J. (1988). Statistical Power for the Social Sciences.

[79] Else-Quest N.M., Hyde J.S., Goldsmith H.H., Van Hulle C.A. (2006). Gender differences in temperament: A meta-analysis. Psychol. Bull..

[80] Lindberg S.M., Hyde J.S., Petersen J.L., Linn M.C. (2010). New trends in gender and mathematics performance: A meta-analysis. Psychol. Bull..

[81] Katzman S., Alliger G.M. (1992). Averaging untransformed variance ratios can be misleading: A comment on Feingold. Rev. Educ. Res..

[82] Hedges L.V., Friedman L. (1993). Gender differences in variability in intellectual abilities: A reanalysis of feingold’s results. Rev. Educ. Res..

[83] Borenstein M., Hedges L.V., Higgins J.P.T., Rothstein H.R. (2009). Introduction to Meta-Analysis.

[84] Higgins J.P.T., Thompson S.G., Deeks J.J., Altman D.G. (2003). Measuring inconsistency in meta-analyses. BMJ.

[85] Leung J.T.Y., Shek D.T.L. (2019). The influence of parental expectations and parental control on adolescent well-being in poor Chinese families. Appl. Res. Qual. Life.

[86] Shek D.T.L. (2008). Parental behavioral control and parent-child relational quality predictors of perceived parental knowledge in Chinese adolescents in Hong Kong. Am. J. Fam. Ther..

[87] Shek D.T.L. (2007). Perceived parental behavioral control and psychological control in Chinese adolescents in Hong Kong: A replication. Adolescence.

[88] Leung J.T.Y., Shek D.T.L. (2012). Parental differences in family processes in Chinese families experiencing economic disadvantage. Multidiscip. J. Gend. Stud..

[89] Lau S., Cheung P.C. (1987). Relations between Chinese adolescents’ perception of parental control and organization and their perception of parental warmth. Dev. Psychol..

[90] Chua A. (2011). Battle Hymm of the Tiger Mother.

[91] Bach D., Christensen S. (2017). Battling the tiger mother: Pre-school reform and conflicting norms of parenthood in Singapore. Child. Soc..

[92] Van Lissa C.J., Keizer R., Van Lier P.A.C., Meeus W.H.J., Branje S. (2019). The role of fathers’ versus mothers’ parenting in emotion-regulation development from mid–late adolescence: Disentangling between-family differences from within-family effects. Dev. Psychol..

[93] Fleming M. (2005). Gender in adolescent autonomy: Distinction between boys and girls accelerates at 16 years of age. Electron. J. Res. Educ. Psychol..

[94] Ahmed I., Sutton A.J., Riley R.D. (2012). Assessment of publication bias, selection bias, and unavailable data in meta-analyses using individual participant data: A database survey. BMJ.

[95] Shek D.T.L. (2014). Applied research in quality of life (ARQOL): Where are we and issues for consideration. Appl. Res. Qual. Life.

